# The molecular landscape of adult diffuse gliomas and relevance to clinical trials

**DOI:** 10.18632/oncotarget.26750

**Published:** 2019-03-05

**Authors:** Patrick J. Cimino, Eric C. Holland

**Affiliations:** Patrick J. Cimino: Department of Pathology, Division of Neuropathology, University of Washington School of Medicine, Seattle, WA, USA; Eric C. Holland: Division of Human Biology, Fred Hutchinson Cancer Research Center, Seattle, WA, USA

**Keywords:** glioblastoma, astrocytoma, biomarkers, clinical trials, copy number variation

Diffuse gliomas comprise the most common primary malignant intracranial neoplasms in adults. In 2016, the World Health Organization (WHO) announced a new classification system for diffuse gliomas that integrates histomorphology with genetic alterations and yields a more predictive system for clinical outcome than histopathology alone [1]. Point mutations in *isocitrate dehydrogenase 1/2* (*IDH1/2*) are the main genetic stratifier in diffuse gliomas, and mutational status indicates a significantly prolonged survival in diffuse gliomas [2]. Looking beyond IDH status, our group has previously shown that a targeted panel of copy number alterations in six genomic regions is prognostic for diffuse glioma, and further stratify IDH-mutant and IDH-wildtype astrocytic gliomas, including glioblastoma [3]. For IDH-mutant diffuse astrocytic gliomas, prognostic copy number alterations include: *CDK4* amplification, *CDKN2A* deletion, and loss of chromosome 14. Furthermore, the individual prognostic significance of *CDK4* amplification and/or *CDKN2A* deletion in IDH-mutant diffuse astrocytic gliomas has been confirmed by other groups [4, 5]. For IDH-wildtype glioblastoma, prognostic copy number alterations include: *CDK4/MDM2* co-amplification, gain of chromosome 1, and gain of chromosome 19. These copy number alterations form specific copy number subtypes, which were originally discovered in The Cancer Genome Atlas (TCGA) dataset, and were validated in a second large general population cohort, the German Glioma Network (GGN) [3].

Given the implications of copy number alteration stratification in the context of IDH mutational status, our group sought to explore the distribution of prognostic copy number subtypes in clinical trial and surgical recurrence cohorts, in order to determine whether or not selection biases occur with trial enrollment [6]. One cohort examined in this study came from a randomized phase II ARTE trial, which examined the effects of radiation both with and without bevacizumab on newly diagnosed glioblastoma in the elderly population [7]. The other cohort examined was from an international collection of paired initial and recurrent glioblastoma, where inclusion required that the patient survived long enough, and was deemed appropriate, to require a second surgery at first recurrence [8]. Indeed, both the medical trial and recurrent surgical cohorts showed a significant distribution shift with enrichment for better performing copy number alteration subtypes [6]. Further, comparison of multidimensional scaling of complete copy number and whole exome sequencing of the surgical cohort relative to the TCGA dataset predicts a population of nearly half of all glioblastoma patients that do not survive long enough to receive a second tumor resection. While data from these glioblastoma trial cohorts spawn intriguing conclusions, additional glioblastoma clinical trial cohorts need to be evaluated to confirm and extend these findings of copy number selection biases. Furthermore, it will be important to investigate lower-grade IDH-mutant diffuse astrocytomas in the clinical trial population to see if similar biases for these copy number subtypes exist.

With what we now know about IDH mutations dichotomizing survival amongst diffuse gliomas, in the modern day it is unimaginable that clinical trials going forward would not stratify patients by IDH-mutational status. Just as clinical trials would not be performed without knowing IDH mutational status, prognostic copy number alterations may be considered in the same family of molecular biomarkers and should be examined with clinical trial enrollment. At a minimum, we would advocate for at least a minimal panel of targeted copy number profiling to inform enrollment glioma trials. Such knowledge would allow for even distribution of copy number alterations across clinical trial arms. Importantly, this stratification would allow for identification of copy number subtype specific therapeutic strategies and efficacy, which are especially needed in the most aggressive subtypes. Overall, copy number biomarker-driven clinical trial stratification has the potential to reduce trial cost, increase testing efficiency, and develop molecular signature specific therapies for subsets of adult diffuse gliomas.
